# Improvements of surgical techniques in a rat model of an orthotopic single lung transplant

**DOI:** 10.1186/2047-783X-18-1

**Published:** 2013-01-07

**Authors:** Haizhou Guo, Jun Nie, Kai Fan, Zhikun Zheng, Xinwei Qiao, Jinsong Li, Jianjun Wang, Ke Jiang

**Affiliations:** 1 Department of Thoracic Surgery, Union Hospital, Tongji Medical College, Huazhong University of Science and Technology, No.1277, Jiefang Avenue, Wuhan, Hubei Province, 430022, P. R. China; 2 Department of Thoracic Surgery, The First Affiliated Hospital, Zhengzhou University, No.1, Jianshe East Road, Zhengzhou, Henan Province, 450052, P. R. China

**Keywords:** Lung transplantation, Surgical technique, rat

## Abstract

**Background:**

Rats are widely used in modeling orthotopic lung transplantation. Recently the introduction of the cuff technique has greatly facilitated the anastomosing procedure used during the transplant. However, the procedure is still associated with several drawbacks including twisting of blood vessels, tissue injury and the extensive time required for the procedure. This study was performed to optimize the model of rat lung transplantation (LT) with the cuff technique.

**Methods:**

A total of 42 adult Lewis rats received orthotopic LT with our newly modified procedures. The modified procedures were based on the traditional procedure and incorporated improvements involving orotracheal intubation; a cuff without a tail; conservative dissection in the hilum; preservation of the left lung during anastomosis; successive anatomizing of the bronchus, the pulmonary vein, and the pulmonary artery; and one operator.

**Results:**

Transplants were performed in 42 rats with a successful rate of 95.23% (40/42). The mean duration for the complete procedure was 82.93 ± 14.56 minutes. All anastomoses were completed in one attempt without vessel laceration, twisting or angulation. In our study, two animals died within three days and one animal died ten days after the operation. All grafts were well inflated with robust blood perfusion and functioned normally as demonstrated by blood gas analysis.

**Conclusions:**

We have developed a modified orthotopic LT technique that can be easily performed while overcoming major drawbacks. The modified technique has many advantages, such as easy graft implanting, shortened operation time, fewer complications and high reproducibility.

## Background

Lung transplantation (LT) is an established therapy for a variety of end-stage pulmonary diseases. According to the report from *The Registry of the International Society for Heart and Lung Transplantation* (ISHLT) in 2010, more than 2700 lung and heart-lung transplantations are performed annually [1]. The overall survival rate after transplantation is currently 79% at one year and about 52% at five years [1]. However, most immune and non-immune mechanisms that complicate the following pathophysiological processes remain poorly understood. Accordingly, many experimental LT models are employed to develop novel preventative and therapeutic strategies [2-6].

Orthotopic LT in an animal model is a procedure that simulates human LT, including rejection, infection and ischemia/reperfusion processes, which can offer much more information for transplantation research. By far, the rat has been one of the more promising candidates employed for LT since first performed in 1971 [7], especially after Mizuta’s cuff technique and a variety of its modifications were introduced in 1989 and later [6,8-11]. We previously established a rat LT model with a modified cuff technique. In our experience there were several disadvantages associated with this procedure, such as the twisting of blood vessels or bronchus and the time required. Recently, we attempted to optimize and modify the previously established cuff model. Our improvements in procedure allow easy handling, a shortened operation time, fewer complications and high reproducibility.

## Methods

### Animals

This study was approved by the Institutional Animal Research Committee of Union Hospital, Tongji Medical school, Huazhong University of Science and Technology. All animals received adequate care in accordance with the National Institutes of Health Guide for the Care and Use of Laboratory Animals. Male inbred Lewis rats were purchased from Hunan Slac Laboratory Animal Company, Ltd (Changsha,Hunan Province, P.R. China). Eight-to-ten-week-old animals weighing 250 to 300 g were used as both donors and recipients. A total of 42 orthotopic LTs were performed in the study.

### Surgical technique

All surgical procedures were performed with sterile techniques in the barrier system of Huazhong University of Science and Technology. An SXP-1C operating microscope with 10x magnification (Shanghai Medical Instruments Company, Ltd, Shanghai, P.R. China.) was used for both donor and recipient operations. Animals were anesthetized with an intraperitoneal injection of sodium pentobarbital (50 mg/kg, Sigma-Aldrich Chemical Shanghai, P.R. China.). Rats were orotracheal intubated with a 16 G angiocatheter and were connected to a ventilator (Shanghai Alcott Biotech Company, Ltd., Shanghai, P.R. China) for respiration assistance with room air and a tidal volume of 10 mL/kg at 85 breaths/minute.

The donor rat was placed in a supine position and a median laparo-sternotomy was performed to expose heart and lungs. The left atrial appendage was cut and lungs were flushed with 20 ml cold (4°C) heparinized LPD (Perfadex™, Addmedica, Paris, France) from the root of the pulmonary artery. The semi-inflated left lung was harvested at end-tidal volume. Its bronchus, pulmonary vein and pulmonary artery were passed through a 14 G, 16 G, and 18 G cuff without tail respectively, with their proximal ends being everted over the cuff and circumferentially fixed.

**Figure 1:**
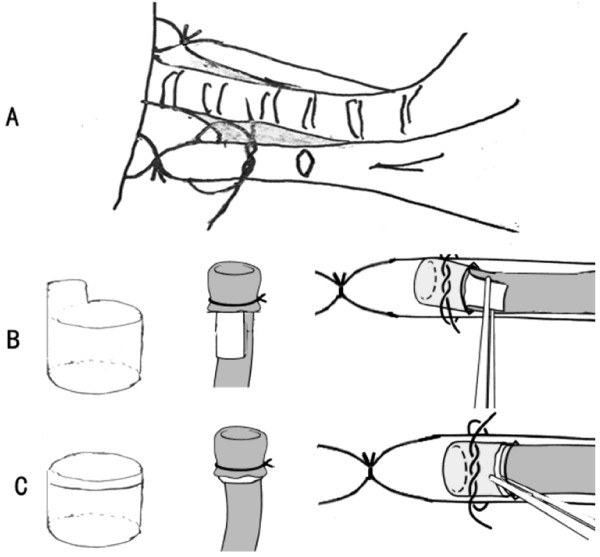
**Improvements on cuff technique. (A)** Conservative dissection in hilum. **(B)** Traditional cuff and implantation. **(C)** Modified cuff and implantation.

Recipients were placed in the right lateral decubitus. Left thoracotomy was performed through the fifth intercostal space and the left lung was retracted laterally out of the thoracic cavity to expose the hilum. The beginning of the left pulmonary artery and pulmonary vein were separated from the bronchus. Thus freeing spaces between them were acquired, where the blood flow was occluded and the anastomosis was finished. Because the pulmonary vein was vulnerable to laceration, dissection was carried out as close to mediastinum as possible and the overlying connective tissues were preserved to antagonize tension and to protect stomas. The three structures in the hilum were individually blocked with slip knots and 7–0 nylon circumferential ligatures were positioned loosely around them. Distal from them, short incisions were made on the anterior wall of the bronchus and the vessels. From which, bronchus, vein and artery cuffs of the donor lung were placed in sequence into the equivalent recipient structures and secured with prepared ligatures (Figure 1A).

The whole procedure was a modification of the traditional method. The differences between the procedures are listed in Table 1.

**Table 1:** Improvements of surgical technique

Improvement	Traditional procedure	Modified procedure
Intubation	Tracheotomy and intubation with 14 G angiocatheter	Orotracheal intubation with 16 G angiocatheter
Cuff	Cuff with tail (Figure 1B)	Cuff without tail (Figure 1C)
Left lung of recipient	Removed to make more room in thoracic cavity for anastomosis	Preserved and retracted laterally until anastomosis was completed
Dissection in hilum	The full length of pulmonary artery and the pulmonary vein trunk as well as the left main bronchus were thoroughly isolated	Conservative dissection was performed. Less than one third of each of these were freed (Figure 1A)
Anastomosing order	The pulmonary vein, the bronchus and the pulmonary artery	The bronchus, the pulmonary vein and the pulmonary artery
Operator	Two surgeons	Single surgeon

### Vital signs

We sampled three LTs to acquire the circulation parameters during operation. The recipient rat was placed in a supine position. Skin in the right groin was prepared and incised. The right common femoral artery was isolated and controlled by arterial clamp. A 24 G angiocatheter (BD Medical Company, Lt, Suzhou, Jiangsu Province, P.R. China)was used to access the artery under visualization and was connected to a transducer for blood pressure measuring. Blood pressure was continuously monitored and recorded by PowerLab data acquisition systems (ADinstruments,Shanghai, P.R. China). Data were processed and analyzed with LabChart Pro software (ADinstruments).

### Functional graft assessment

Blood gas analysis was performed to assess the lung graft function. At the time of sacrifice on postoperative day 7, the right lung pulmonary hilum was occluded for ten minutes. Two samples of blood were drawn from the left atrium pre-occlusion and post-occlusion respectively. PaO_2_ and PaCO_2_ were measured on a FiO_2_ of 0.21. In addition, photographs were taken on day 7 after lung transplantation to evaluate changes in the chest cavity.

### Histology

Transplanted lungs were harvested after pulmonary flushing with normal saline, fixed in 4% paraformaldehyde, sectioned and stained with hematoxylin and eosin. Grading for rejection pathology was performed by a pathologist using standard criteria developed by the Lung Rejection Study Group [12].

### Statistics

Statistical analysis was performed with SPSS version 13.0 software (Chicago, IL, USA). Data were reported as means ± SD and compared with paired *t* test. *P* values less than 0.05 were considered statistically significant.

## Results

### Outcomes

We performed 42 transplants with a success rate of 95.23%. Two animals died of bleeding and pulmonary vein injury during the operation. Another two animals died within three days after the operation and one died on postoperative day 10. All remaining animals were euthanized on the day of our predetermined end point of the study. The whole procedure was performed in 82.93 ± 14.56 minutes and the relevant periods of procedure times are listed in Table 2. All the anastomoses were completed in one attempt. Some common complications such as vessel laceration, twisting and angulation that occur in traditional procedures were not observed.

**Table 2:** Relevant times in orthotopic lung transplantation (mean ± SD)

Procedure	Minutes
Donor procedure	24.75 ± 7.47
Graft preparation	18.70 ± 5.65
Recipient procedure	24.65 ± 4.75
(Anastomosis time)	(15.23 ± 2.25)
Other	14.83 ± 4.87
Total procedure	82.93 ± 14.56

### Vital signs

**Figure 2:**
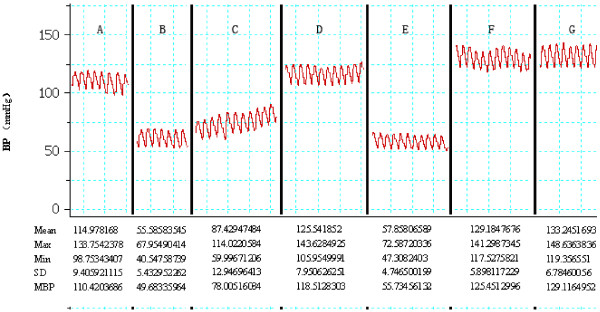
**Dynamics of blood pressure and electrocardiogram during operation. (A)** Preoperative. **(B)** Mechanical ventilation begins. **(C)** Mechanical ventilating. **(D)** Intra-operative. **(E)** Left hilum blocked. **(F)** Graft reperfused. **(G)** Postoperative.

Generally, vital signs of the recipients were stable for the entire operation procedure, with the exception of two transient hypotensive episodes. These were observed at the beginning of mechanical ventilation but subsequently normal pressure returned gradually and the period of hilum blocking recovered with the reperfusion of graft (Figure 2).

### Functional graft assessment

**Figure 3:**
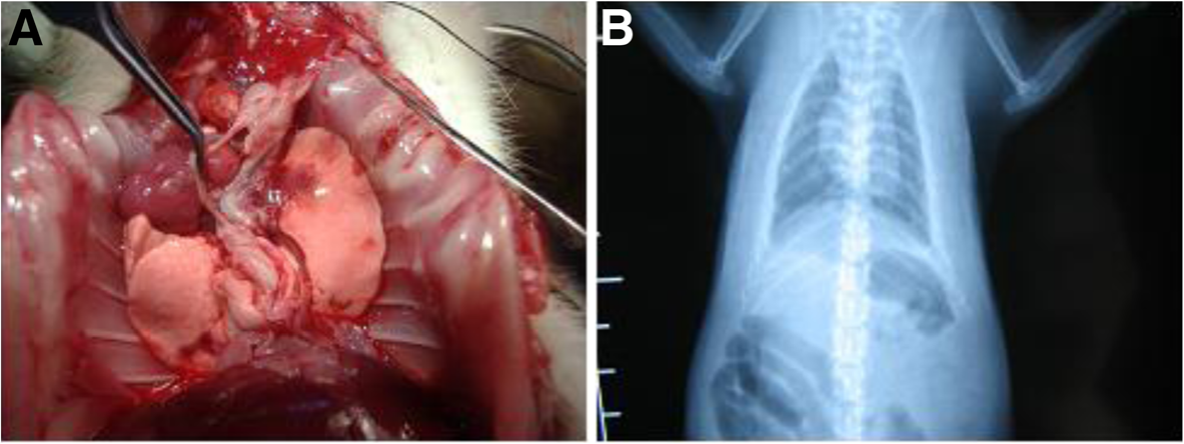
Postoperative recipient at day 7, showing well-inflated graft and potent function.

Exploration on postoperative day 7 showed that the grafts were well inflated with a robust blood supply (Figure 3). Postoperative chest radiography illustrated the graft was well inflated and blood gas analysis demonstrated normal function (Table 3).

**Table 3:** O**xygenation capacity of grafts on postoperative day 3 (n = 8, FiO2 = 0.21)**

Ventilation	PaO_2_		PaCO_2_	
	Mean	SD	Mean	SD
Double lung	71.96	4.75	41.24	4.21
Single lung	68.68	5.58	43.70	3.70
*P*	0.12		0.07	

### Histology

**Figure 4:**
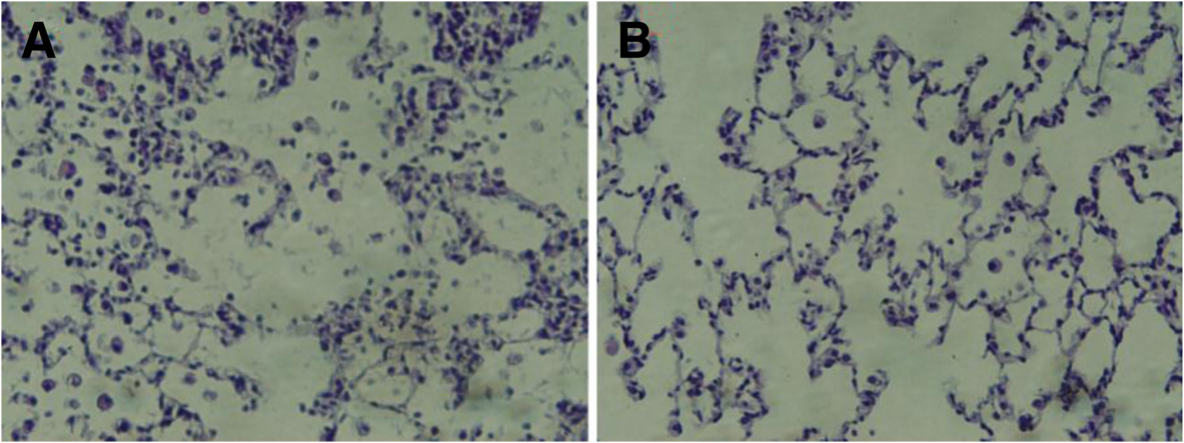
**Representative ischemia-reperfusion injury following transplantation. (A)** Three days after operation, transplant recipient manifests widened alveolar septa, presence of multiple alveolar macrophages and neutrophil, and obvious edema. **(B)** Seven days after operation, transplant recipient manifests nearly normal septa, very few alveolar macrophages and sparse edema. (hematoxylin and eosin. 400x).

The hematoxylin and eosin (H.E) stain of recipient lung section showed lung injury of alveolar exudation, septa edema and interstitial infiltration on postoperative day 3. However, those lesions disappeared on postoperative day 7, indicating recovery from ischemia-reperfusion lung injury sustained in the first 3 days posttransplant (Figure 4).

## Discussion

Orthotopic LT in rats challenges surgeons greatly for its all-around demands, such as well sensation of micromanipulation under microscope, good performance during operation, precise collaboration between surgeons and intensive care after transplant. Therefore, a simple and easy-to-perform model with high reproducibility is of great importance. Although the Mizuta’s cuff technique greatly facilitates anastomosis during the operation, many drawbacks remain. For example, vessels and bronchus are likely to twist during the procedure, and the pulmonary vein is vulnerable to laceration during anastomosis. Our experience shows that the modified technique we adopted has fewer complications and improved outcomes. Compared with our previous study, the whole procedure could be executed easily by one instead of two surgeons. Although this reflects the learning curve of the model to some extent, its high reproducibility and low incidence of mortality indeed illustrate the rationality of these improvements.

Minitracheostomy is a reliable method of guaranteeing undisturbed intubation and ventilation. However, it is time-consuming and traumatic. For those laboratory animals that will be euthanatized at the end of experiment, it is an acceptable method. But for the study of lung transplantation, acute rejection often happens within the first postoperative week and studies of chronic rejection require a longer observation period. Infection, scarring and even stenosis in trachea following tracheostomy might confound the interpretation of experimental data due to its potential impact on lung function of the recipient. Another disadvantage of tracheostomy is the inconvenience of postoperative care. We previously observed that rats undergoing tracheostomy often had difficulty breathing as demonstrated by inspiratory and expiratory stridor, which accompanied a high mortality during the first three days after operation. But in this study, animals demonstrated steady breathing and recovered rapidly during the early postoperative period. Accordingly, we translated tracheostomy into orotracheal intubation after operation and intensive postoperative care was needed for those animals. Clear exposure of the glottis is the most important step in the orotracheal intubation. According to our experience, as long as the rat head is flexed back to keep the chest, neck and lower jaw in a straight line, which provides a moderate stretch to the rat tongue, the glottis of the rat is easily visualized.

A traditional cuff consists of a body 1.5 mm in length and a tail 1 mm in length [8]. Its tail is designed for the convenience of cuff setting and inserting. However, in addition to the foreign-body reaction and fibrous formation caused by this kind of cuff, it also occupies more space. Therefore, a relatively longer length of the bronchus or vascular vessels needs to be dissected during the operation, otherwise the lung tissue in the hilum would be compressed and even injured by the cuff tail. Furthermore, when the vascular cuff is prepared, its orientation should be taken into consideration during anastomosis. Vessel twisting or angulation related to inappropriate placement of cuff could affect the flow of pulmonary circulation, which then would cause graft ischemia or congestion. In this study, the cuff tail was removed, but we could hold any point of the cuff body and make slight adjustments when necessary, which effectively avoided the twisting of vessels or bronchus without increasing difficulty of implantation. Because our cuffs were cut from smooth angiocatheters, everted tissue on the surface was inclined to slip out because of tension from the graft during delivery. Although roughening the cuff with sandpaper and everting more tissue could prevent detachment, the apical segmental bronchus and left upper pulmonary vein would be affected because they originated from the proximal trunk. Focal imbalance of ventilation/perfusion would in turn occur for the reason of their stenosis or occlusion. To overcome these disadvantages, we created a nick 0.2 mm to one end, where cuffs were circumferentially ligated. Thus, only a small amount of tissue is required to be everted but it remains well-fixed.

The main cause of death in operation is hemorrhage, which is most often caused by the tearing of the pulmonary vein. As a result, preventive procedures must be performed to avoid this disastrous event. In our previous study, we routinely removed the native lung to make sufficient space in the chest cavity and we preserved enough length of proximal blood vessel for anastomosis. The main procedures were done in the small chest cavity, which is inconvenient. Furthermore, a pull-out force should be exerted for exposition and cuff inserting, which cannot be done without assistance. During these procedures, thin-walled vessels may be torn due to carelessness or miscoordination between surgeons, especially in the area adjacent to the lung parenchyma. Accordingly, sophisticated techniques, repeated practice, and precise collaboration are required. During this new procedure, the native lung was preserved and laterally retracted out of the thoracic cavity until anastomosis was finished. Consequently, the mediastinum shifted to the left and the hilum was exposed superficially. Those modifications allowed for better visualization and made dissection and anastomosis easier.

Among the three hilar structures, the pulmonary vein is vulnerable as a result of its anatomic character [10]. Preserving its overlying connective tissues to antagonize tension during anastomosis could help to prevent accidental laceration and protect stomas. Our experience showed that conservative dissection near the mediastinum and the creation of these two small free spaces yielded sufficient space for ligation and anastomosis. Accordingly, it is unnecessary to thoroughly isolate the hilum during recipient procedure. In addition, successive anastomosing of artery, bronchus and vein respectively, or in a reverse order with the intention of protecting the vein, was adopted by different researchers [8,13]. But those procedures have a potential risk of vessel twisting. To improve this, we anastomosed the bronchus first, followed by vein, and then artery. The advantage of this modification is that the wide lumen and thick wall of bronchus is convenient for cuff inserting and fixation. The circumferential ligature between cartilage rings on the cuff will secure the firm fixation of the graft, enabling it to resist tension during the following procedure. More importantly, the anastomosed bronchus constitutes a major part of the three-dimensional structure of hilum, which was used to guide insertion of vessels. The pulmonary vein and artery cuff were easily placed into their equivalent structure while in their natural state. Therefore, no twisting occurred in our study.

In order to prevent injury to the bronchial and vascular wall, an incision is made only on the anterior wall of the one-third of the bronchus and vessel of the recipient. If the incision is not long enough, the surgeon can cut the incision into a ‘├’shape towards the recipient hilum. The operator uses his left hand to lift the front wall of the recipient bronchial-vascular structures to expose the lumen and uses his right hand to hold the donor’s bronchial-vascular structures, inserting them into the recipient’s lumen through the incision in the wall mediastinally. Thus, this superficial modification makes it easy for the surgeon to perform LT without an assistant.

## Conclusions

In conclusion, our modified procedure could further facilitate graft implanting, reduce operating time and increase reproducibility in a rat LT model.

## Competing interests

The authors report no conflict of interest to disclose.

## Authors’ contributions

Haizhou Guo performed 31 LTs and drafted the manuscript. Jun Nie participated in the design of the study and helped to draft the manuscript. Kai Fan participated in the design of the study and performed 11 LTs. Zhikun Zheng carried out the HE stain and helped to draft the manuscript. Xinwei Qiao carried out blood gas analysis, blood pressure monitoring and was in charge with the perioperative management. Jinsong Li participated in the design of the study and performed the statistical analysis. Jianjun Wang participated in its design and supervised of the research group. Ke Jiang conceived of the study, and participated in its design and coordination and helped to draft the manuscript. All authors read and approved the final manuscript.

## References

[B1] Christie JD Edwards LB Kucheryavaya AY Aurora P Dobbels F Kirk R Rahmel AO Stehlik J Hertz MI The Registry of the International Society for Heart and Lung Transplantation: twenty-seventh official adult lung and heart-lung transplant report-2010 J Heart Lung Transplant 2010 29 1104 1118 10.1016/j.healun.2010.08.004 20870165

[B2] Sato M Keshavjee S Liu M Translational research: animal models of obliterative bronchiolitis after lung transplantation Am J Transplant 2009 9 1981 1987 10.1111/j.1600-6143.2009.02770.x 19663891

[B3] Jiang X Khan MA Tian W Beilke J Natarajan R Kosek J Yoder MC Semenza GL Nicolls MR Adenovirus-mediated HIF-1alpha gene transfer promotes repair of mouse airway allograft microvasculature and attenuates chronic rejection J Clin Invest 2011 121 2336 2349 10.1172/JCI46192 21606594 PMC3104770

[B4] Zanotti G Casiraghi M Abano JB Tatreau JR Sevala M Berlin H Smyth S Funkhouser WK Burridge K Randell SH Egan TM Novel critical role of Toll-like receptor 4 in lung ischemia-reperfusion injury and edema Am J Physiol Lung Cell Mol Physiol 2009 297 L52 L63 10.1152/ajplung.90406.2008 19376887 PMC2711808

[B5] Zhang R Wang Z Wang H Song H Zhang N Fang M Optimal pulmonary artery perfusion mode and perfusion pressure during cardiopulmonary bypass J Cardiovasc Surg (Torino) 2010 51 435 442 20523296

[B6] Okazaki M Krupnick AS Kornfeld CG Lai JM Ritter JH Richardson SB Huang HJ Das NA Patterson GA Gelman AE Kreisel D A mouse model of orthotopic vascularized aerated lung transplantation Am J Transplant 2007 7 1672 1679 10.1111/j.1600-6143.2007.01819.x 17511692

[B7] Asimacopoulos PJ Molokhia FA Pegg CA Norman JC Lung transplantation in the rat Transplant Proc 1971 3 583 585 4937944

[B8] Mizuta T Kawaguchi A Nakahara K Kawashima Y Simplified rat lung transplantation using a cuff technique J Thorac Cardiovasc Surg 1989 97 578 581 2648080

[B9] Zhai W Ge J Inci I Hillinger S Markus C Korom S Weder W Simplified rat lung transplantation by using a modified cuff technique J Invest Surg 2008 21 33 37 10.1080/08941930701834114 18197532

[B10] Jungraithmayr WM Korom S Hillinger S Weder W A mouse model of orthotopic, single-lung transplantation J Thorac Cardiovasc Surg 2009 137 486 491 10.1016/j.jtcvs.2008.10.007 19185174

[B11] Goto T Kohno M Anraku M Ohtsuka T Izumi Y Nomori H Simplified rat lung transplantation using a new cuff technique Ann Thorac Surg 2012 93 2078 2080 10.1016/j.athoracsur.2012.01.096 22632516

[B12] Stewart S Fishbein MC Snell GI Berry GJ Boehler A Burke MM Glanville A Gould FK Magro C Marboe CC McNeil KD Reed EF Reinsmoen NL Scott JP Studer SM Tazelaar HD Wallwork JL Westall G Zamora MR Zeevi A Yousem SA Revision of the 1996 working formulation for the standardization of nomenclature in the diagnosis of lung rejection J Heart Lung Transplant 2007 26 1229 1242 10.1016/j.healun.2007.10.017 18096473

[B13] Ke J Lin C Wang J Li J Nie J Heme oxygenase-1 expression in rats with acute lung rejection and implication J Huazhong Univ Sci Technolog Med Sci 2009 29 84 87 10.1007/s11596-009-0118-0 19224170

